# Common genetic mechanisms between obesity and COVID-19 severity: unravelling pleiotropic loci and biological pathways

**DOI:** 10.1098/rsos.252355

**Published:** 2026-08-12

**Authors:** Giulia Souza da Costa, Cibele E. Bandeira, Nicolas Pereira Ciochetti, Isabella F. Temoteo, Bianca Rodrigues Martinello, Thais Martins-Silva, Raissa Nunes Dos Santos, Diego L. Rovaris, Marilu Fiegenbaum, Lucia Campos Pellanda, Luciana Tovo-Rodrigues, Júlia Pasqualini Genro

**Affiliations:** ^1^ Graduate Program in Biosciences, Federal University of Health Sciences of Porto Alegre, Porto Alegre, Brazil; ^2^ Department of Basic Health Sciences, Federal University of Health Sciences of Porto Alegre, Porto Alegre, Brazil; ^3^ Biomedicine School, Federal University of Health Sciences of Porto Alegre, Porto Alegre, Brazil; ^4^ Graduate Program in Pediatrics, Federal University of Health Sciences of Porto Alegre, Porto Alegre, Brazil; ^5^ University of São Paulo, Brazil; ^6^ Federal University of Pelotas, Pelotas, RS, Brazil; ^7^ Graduate Program in Epidemiology, Federal University of Pelotas, Pelotas, Brazil

**Keywords:** COVID-19, obesity, pleiotropy, genetic overlap, genetic susceptibility, GWAS, conjFDR, PheWAS, BMI

## Abstract

COVID-19 and obesity are complex conditions marked by immune and metabolic dysfunction, with the former still ranking among the leading causes of death from infectious diseases worldwide and the latter reaching pandemic proportions. Clinical evidence consistently shows that obesity increases the risk of severe COVID-19, yet the biological mechanisms underlying this association remain unclear. Given their physiological and clinical overlap, they may share genetic pathways. We investigated genetic variants jointly associated with body mass index (BMI) and COVID-19 using publicly available genome-wide data. A conjunctional false discovery rate (conjFDR) approach identified shared variants between BMI and three COVID-19 phenotypes: infection, hospitalization and very severe respiratory illness. Functional annotation and pathway enrichment analyses were performed to explore the biological context of these variants, followed by a phenome-wide association study (PheWAS) to characterize pleiotropy. Shared variants were enriched in immune, metabolic and hormonal signaling pathways, including metal ion transport and glycosylation. The overlap with BMI was strongest for hospitalized and severe cases, suggesting common mechanisms underlying disease progression rather than infection. These findings suggest a biologically meaningful genetic overlap between obesity and COVID-19 severity, highlighting pleiotropy as a key feature in complex disease interactions and potential shared therapeutic targets.

## 1. Introduction

Five years after the onset of the pandemic, COVID-19 remains a challenge for global health systems. Although the disease has reached a state of relative stability, it has never ceased to cause infections and deaths and is currently the second leading cause of death among infectious diseases worldwide [1]. Regrettably, stark disparities in vaccine distribution are still evident: less than half the population of low-income countries has received even a single vaccine dose, and their case fatality rate is more than double the global average [2].

The different responses to SARS-CoV-2 among the population have highlighted the multifactorial nature of COVID-19. While some infected individuals are asymptomatic or develop mild flu-like symptoms, others experience severe symptoms, leading to impairment of vital functions [3]. This heterogeneity in response to the virus suggests a complex phenotype in which genetics plays a role in disease progression. Dozens of genome-wide association studies (GWAS) have shown that host genetics is indeed relevant to the clinical course of the infection [4,5]. However, the presence of multiple clinical variables associated with severe outcomes, many of which are themselves complex and multifactorial diseases, such as COVID-19, points to a genetic contribution that remains beyond the scope of existing genetic studies.

According to the World Health Organization, obesity represents another ongoing pandemic, with more than one billion people living with the disease worldwide [6,7]. It has been consistently associated with the most severe forms of COVID-19, increasing the likelihood of hospitalization, intensive care unit admission, mechanical ventilation and mortality. Approximately 80% of infected patients admitted to intensive care units with respiratory failure are overweight or obese [8,9]. Despite the strong association between severe COVID-19 and obesity, the mechanisms underlying this relationship remain poorly explored. Considering that these two conditions are not only strongly linked in clinical settings but also represent complex and multifactorial diseases, they may share common genetic pathways. Since the pathophysiology of both severe COVID-19 and obesity involves an exacerbated inflammatory response, this further supports the hypothesis that their genetic etiology may be interconnected, particularly through immune and inflammatory pathways [10].

To date, large-scale GWAS, including meta-analyses from the COVID-19 Host Genetics Initiative and the GenOMICC consortium, have identified over 60 genes or genomic loci associated with increased risk of severe COVID-19 [4,5,11,12]. Similarly, large-scale GWAS for body mass index (BMI) have identified numerous loci associated with this phenotype, and summary-level data are publicly available [13]. While Mendelian randomization has been used to investigate potential causal relationships between obesity and COVID-19 outcomes [14–16], only one study has examined the genetic overlap between these two conditions, using BMI as a genetic proxy for obesity [17]. Although shared loci and enriched immune-related pathways were identified, no study has employed an approach specifically designed to detect shared genetic architecture while also assessing differences between susceptibility and severity and providing a detailed phenotypic characterization of pleiotropy.

In this study, we adopt an integrative analytical approach to investigate loci shared between BMI and COVID-19 outcomes. Using publicly available summary statistics from large-scale GWAS, we applied the conjunctional false discovery rate (conjFDR) method, which improves statistical power by leveraging cross-trait associations beyond traditional genetic correlation. This hypothesis-free strategy enables the identification of single nucleotide polymorphisms (SNPs) jointly associated with BMI and COVID-19 phenotypes. Following SNP-to-gene annotation, we conducted functional enrichment analyses across curated biological pathways. To better characterize the pleiotropy, we explore other phenotypes associated with each SNP identified using a phenome-wide association studies (PheWAS) strategy.

## 2. Material and methods

### 2.1. Genome-wide association studies summary statistics

The summary statistics for COVID-19 were obtained from the COVID-19 Host Genetics Initiative (https://www.covid19hg.org/)—release 7. Three GWAS of COVID-19 from European population were included: (i) COVID-19 versus population, with 122 616 cases, 2 475 240 controls and 14 496 978 variants; (ii) very severe respiratory confirmed COVID-19 versus population, including 13 769 cases, 1 072 442 controls and 12 174 527 variants; and (iii) hospitalized COVID-19 versus population, with 32 519 cases, 2 062 805 controls and 12 469 431 variants.

In this study, BMI was used as a genetic proxy for obesity, as large-scale GWAS summary statistics are currently available for this phenotype. The summary statistics for BMI were obtained from the GIANT consortium, which included 681 275 subjects and 2 336 269 variants [13]. To minimize potential confounding owing to population stratification and ensure compatibility in linkage disequilibrium (LD) patterns across datasets, the analyses were restricted to the European-ancestry GWAS released by the GIANT consortium and the COVID-19 Host Genetics Initiative.

All summary statistics underwent quality control procedures prior to analysis. We removed duplicate variants and those lacking an rsID, resulting in final datasets comprising: (i) 14 141 478 variants for COVID-19 versus population, representing susceptibility to infection (hereafter referred to as COVIDPOP); (ii) 12 315 684 variants for hospitalized COVID-19 versus population, representing an intermediate level of disease severity (hereafter referred to as HOSPOP); (iii) 12 027 724 variants for very severe COVID-19 versus population, representing the most severe disease outcome (hereafter referred to as SEVPOP); and (iv) 2 336 260 variants for BMI.

### 2.2. Conjunctional false discovery rate

We evaluated the overlap of genetic markers through the conjFDR approach. The conjFDR method is an extension of the conditional false discovery rate (condFDR) method [18,19], which is based on the premise that SNPs with pleiotropic effects have a higher probability of being the causal ones. CondFDR re-ranks the test statistics of a primary phenotype (i.e. COVID-19) by conditioning on SNP associations with a secondary phenotype (i.e. BMI). After switching the primary and secondary phenotypes, conjFDR was determined as the maximum of the two condFDR values.

ConjFDR follows a bottom-up framework. First, all SNPs meeting the significance threshold (conjFDR < 0.05) are identified. These SNPs are then iteratively grouped based on LD (*r*² < 0.1), using an LD matrix derived from the 1000 Genomes Project Phase 3 reference panel. Through this iterative procedure, SNPs in LD with variants already assigned to a given region are progressively incorporated, resulting in the definition of a genomic locus as connected sets of associated variants. Consequently, a locus may contain more than one LD block, as long as these are connected through the underlying LD structure, even if the connections between blocks are relatively weak. Finally, conjFDR applies the FastPrune algorithm, which ranks SNPs according to their statistical significance (−log₁₀ of the FDR values) and retains only the most significant SNP within each block to indicate the lead SNP, as described in https://github.com/precimed/pleiofdr.

### 2.3. Pathway enrichment analysis

We performed functional enrichment analysis with EnrichR, a pathway-analysing tool that compiles 35 different pathway databases [20–22]. First, the lead SNPs identified by conjFDR were annotated to genes (overlapped with the gene if available, or the nearest upstream or downstream gene within a 20 kb window) using SNP-nexus [23]. The resulting list of genes was then analysed for enrichment across Reactome 2022 pathways, Kyoto Encyclopedia of Genes and Genomes (KEGG) 2026 pathway and Gene Ontology Biological Process, Cellular Component and Molecular Function databases.

Pathways were considered significant at an FDR-adjusted *q*-value less than 0.05. FDR correction was performed using the Benjamini–Hochberg procedure applied within each database, as implemented in Enrichr. As this implementation does not control for multiple testing across all databases simultaneously, nor across the different phenotypes analysed, enrichment results were interpreted with caution, and emphasis was placed on biologically consistent findings.

### 2.4. Phenome-wide association study

A PheWAS was performed using the GWAS Atlas platform (https://atlas.ctglab.nl/PheWAS), a curated database that aggregates summary statistics from thousands of GWAS covering a wide spectrum of phenotypes derived from multiple cohorts. As the database integrates results from independent studies, sample sizes vary across traits and correspond to those reported in the original studies. The PheWAS was used to identify robust cross-phenotype associations, and only SNP–trait associations with *P* ≤ 1 × 10^−8^ were considered, corresponding to the conventional genome-wide significance threshold used in GWAS.

To reduce the burden of the PheWAS analysis, we focused only on the lead SNPs identified by conjFDR that were also concordant—that is, those that were: (i) neither associated in the original GWASs for BMI or COVID-19 (i.e. not hits in either phenotype), or (ii) significant in both GWASs (i.e. hits in both phenotypes). SNPs that were hits in only one of the original GWAS, and therefore discordant for their prior associations, were excluded, as their conjFDR significance could be primarily driven by a single trait rather than reflecting true pleiotropy.

## 3. Results

**Figure 1.**
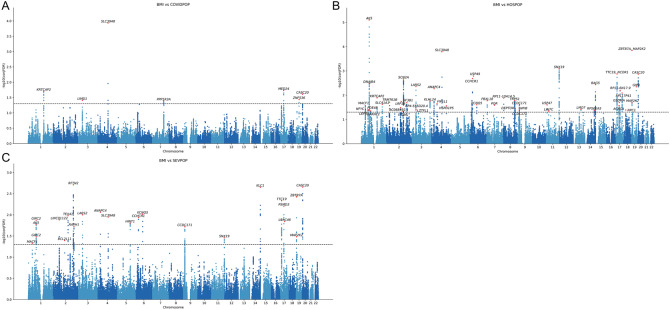
Manhattan plot of conjFDR analyses for (A) BMI and COVID versus population; (B) BMI and hospitalized COVID versus population; and (C) BMI and very severe respiratory confirmed COVID versus population. The *X*-axis displays each chromosome, and the *Y*-axis shows the −log_10_ of the conjFDR *p*-value for each SNP tested. The dashed line indicates the significance threshold (−log_10_(conjFDR < 0.05)). The lead SNPs from significant loci are highlighted in red and labeled with their nearest gene. For those that were not located within any gene, the name of the nearest gene (up or downstream) is shown. For a complete list of the genes, pseudogenes or non-coding regions annotated for these SNPs, see tables 1–3. BMI; COVIDPOP, COVID versus population; HOSPOP, hospitalized COVID versus population; SEVPOP, very severe respiratory confirmed COVID versus population.

ConjFDR analysis revealed an overlap between genetic markers for BMI and COVID-related phenotypes. A total of seven lead SNPs were jointly associated with BMI and COVID-19 infection (COVIDPOP) (figure 1A and table 1; supplementary material, fig. S1), mapping to seven independent loci. For BMI and hospitalized COVID-19 (HOSPOP), 50 lead SNPs were identified (figure 1B and table 2; supplementary material, fig. S1), mapping to 46 independent loci. Finally, for BMI and very severe respiratory confirmed COVID-19 (SEVPOP), 24 lead SNPs were identified (figure 1C and table 3; supplementary material, fig. S1), which represented 21 independent loci. All SNPs that crossed the significance conjFDR threshold (conjFDR < 0.05), regardless of LD structure, are reported in supplementary material tables S1–S3, totaling 28 SNPs for COVIDPOP, 612 for HOSPOP and 226 for SEVPOP.

**Table 1.** Lead SNPs identified by conjFDR for BMI and COVIDPOP.

locus	SNP	chr	pos	A1	A2	Zscore_BMI	Zscore_COVIDPOP	conjfdr_BMI_COVIDPOP	annotated gene
1	rs4276913	1	155131673	G	A	−3.4312	−6.6128	0.0198	*HMGN2P18; KRTCAP2*
2	rs1021124	3	45623774	G	A	−3.3955	4.0141	0.0391	*LIMD1*
3	rs13107325	4	103188709	T	C	9.8875	5.2867	0.0001	*SLC39A8*
4	rs12705894	7	113351252	G	A	6.0992	3.9983	0.0410	*TSRM; PPP1R3A*
5	rs7502514	17	38188844	A	G	−3.7108	−4.2357	0.0185	*MED24*
6	rs33431	19	30939989	C	T	−4.5012	−4.0351	0.0367	*ZNF536*
7	rs2326788	20	6470094	A	G	3.9973	−4.1557	0.0246	*CASC20*

Concordant lead SNPs identified by the conjFDR analysis and subsequently explored in PheWAS analyses are highlighted in grey.

**Table 2.** Lead SNPs identified by conjFDR for BMI and HOSPOP.

locus	SNP	chr	pos	A1	A2	Zscore_BMI	Zscore_ HOSPOP	conjfdr_BMI_ HOSPOP	annotated gene
1	rs2018212	1	39905588	C	T	5.0934	3.7345	0.0272	*MACF1*
2	rs12060189	1	41160281	G	A	−3.0148	−3.7037	0.0387	*NFYC*
3	rs11208648	1	65896612	G	A	3.9243	3.5548	0.0427	*LEPR; LEPROT*
4	rs17451403	1	66387487	T	C	−4.4073	−3.5882	0.0394	*PDE4B*
5	rs9324162	1	77954804	G	A	7.3140	5.7552	0.0000	*AK5*
5	rs4130548	1	78463868	C	T	6.8118	4.4259	0.0033	*DNAJB4; GIPC2*
6	rs6699397	1	91212216	G	A	2.9604	4.2171	0.0446	*BARHL2; RP4-665J23.1*
7	rs4971079	1	155130391	A	G	−3.4060	−4.9985	0.0129	*HMGN2P18; KRTCAP2*
8	rs823154	1	205762406	T	C	−4.0673	−3.7818	0.0241	*SLC41A1*
9	rs11684695	2	29088450	T	G	4.0673	−3.8625	0.0195	*TRMT61B*
10	rs4954388	2	136808142	C	T	−3.0777	3.7841	0.0327	*AC093391.2; AC068492.1*
11	rs1449488	2	142878608	C	T	3.2883	3.9058	0.0181	*LRP1B*
12	rs13023477	2	161111887	T	C	3.5150	3.5144	0.0472	*ITGB6*
13	rs2304013	2	166227050	G	T	−4.2705	−4.5411	0.0022	*SCN2A*
14	rs12151767	2	198274929	G	A	−3.2466	−3.8934	0.0205	*SF3B1*
15	rs2372580	3	44049945	A	G	5.0038	3.6549	0.0333	*RP4-555D20.4; RNU6-367P*
16	rs33807	3	45334692	A	G	3.9216	−4.3437	0.0044	*U3; LARS2*
16	rs12493471	3	45951678	T	C	−2.9963	4.2902	0.0407	*LZTFL1*
18	rs6807489	3	183394462	G	A	−3.5976	−3.8978	0.0176	*KLHL24*
19	rs7664615	4	25448493	A	G	3.6890	5.2973	0.0054	*ANAPC4; RP11-206P5.1*
20	rs2590775	4	54597015	A	G	3.2143	3.9660	0.0224	*FIP1L1; RP11-317M11.1*
21	rs13107325	4	103188709	T	C	9.8864	5.1609	0.0002	*SLC39A8*
22	rs12651029	4	145770167	A	G	−3.3602	−3.7310	0.0275	*HSPD1P5; RP13-539F13.3*
25	rs1265087	6	31109810	A	G	4.1193	4.5546	0.0021	*POLR2LP; CCHCR1*
26	rs6901756	6	41825590	C	T	−4.0462	−4.9773	0.0016	*USP49*
27	rs10943075	6	73719982	G	A	3.3337	3.7691	0.0249	*KCNQ5*
28	rs2966431	7	5552436	A	G	3.9067	3.9141	0.0169	*FBXL18*
29	rs10954732	7	75611149	G	A	3.2983	−3.7546	0.0259	*POR*
30	rs2010390	8	9047178	A	G	4.6970	−4.0013	0.0132	*RP11-10A14.5*
31	rs800591	8	116811386	T	C	−6.1505	−3.9143	0.0169	*TRPS1*
32	rs13260933	8	120907250	G	A	−3.1832	−3.6790	0.0345	*DEPTOR*
33	rs4741388	9	14514366	C	T	3.5976	3.6466	0.0341	*NFIB; RP11-408A13.2*
34	rs3129709	9	15609608	G	A	3.0226	3.6466	0.0428	*CCDC171*
34	rs7045018	9	15773606	A	C	−6.8208	−3.7695	0.0249	*CCDC171*
35	rs10831690	11	11898743	G	T	3.1786	3.8846	0.0248	*USP47*
36	rs4922787	11	27507902	G	T	9.1495	3.5298	0.0454	*RP11-159H22.1; LIN7C*
37	rs7106973	11	130758113	A	G	4.2384	4.7988	0.0008	*SNX19*
38	rs9573671	13	76429311	A	G	3.0179	−4.3030	0.0383	*LMO7*
39	rs1286143	14	91477219	A	G	−4.1533	3.5663	0.0416	*RPS6KA5*
40	rs7148456	14	104028270	C	T	3.8081	4.4490	0.0037	*BAG5; KLC1*
41	rs3785632	17	15945608	C	T	−4.4073	−4.6664	0.0013	*TTC19; NCOR1*
42	rs2121017	17	35537338	A	G	3.2701	3.7458	0.0270	*ACACA*
43	rs3859192	17	38128648	T	C	−3.3757	−3.8500	0.0201	*GSDMA*
44	rs11650043	17	46027852	A	G	−3.6353	−4.2655	0.0064	*RP11-6N17.9*
45	rs12600551	17	65792997	A	G	3.5567	4.1835	0.0082	*RPL17P41; AC006534.1*
46	rs1788820	18	21101944	A	G	7.0504	3.5740	0.0408	*C18orf8; NPC1*
47	rs7254272	19	4069119	A	G	−5.7023	−5.1861	0.0001	*ZBTB7A; MAP2K2*
47	rs350894	19	4115562	A	G	3.2395	4.0678	0.0209	*MAP2K2*
48	rs2334255	19	46186150	T	G	3.7451	4.3974	0.0045	*GIPR*
49	rs17721822	20	6469596	A	G	4.0992	−4.9573	0.0014	*CASC20*

Concordant lead SNPs identified by the conjFDR analysis and subsequently explored in PheWAS analyses are highlighted in grey.

**Table 3.** Lead SNPs identified by conjFDR for BMI and SEVPOP.

locus	SNP	chr	pos	A1	A2	Zscore_BMI	Zscore_ SEVPOP	conjfdr_BMI _SEVPOP	annotated gene
1	rs2484749	1	39758955	A	G	3.8565	3.7241	0.0471	*MACF1*
2	rs7542588	1	77938165	G	T	5.1270	4.1018	0.0168	*AK5*
2	rs12758374	1	78590255	C	A	−4.8399	−4.1793	0.0134	*GIPC2*
2	rs17391694	1	78623626	T	C	8.7602	3.8619	0.0330	*GIPC2; RNFT1P2*
3	rs12713384	2	58623301	G	A	−4.1172	−4.1858	0.0131	*AC007250.4; LINC01122*
4	rs17484848	2	111900560	C	T	3.5354	−3.7833	0.0405	*BCL2L11*
5	rs6721590	2	145612872	G	A	3.9071	4.2585	0.0105	*TEX41*
6	rs6728904	2	198492837	C	T	−4.4148	−4.7261	0.0020	*RFTN2*
7	rs2469953	2	204322298	T	C	−3.9083	−4.0667	0.0187	*RAPH1*
8	rs17576051	3	45449980	G	A	3.6414	−4.5184	0.0100	*LARS2*
9	rs7664615	4	25448493	A	G	3.6871	4.9639	0.0087	*ANAPC4; RP11-206P5.1*
10	rs13107325	4	103188709	T	C	9.8814	4.2346	0.0113	*SLC39A8*
11	rs1549233	5	130417501	C	A	−3.4943	4.1883	0.0157	*RP11-114H7.3; HINT1*
12	rs1265087	6	31109810	A	G	4.1172	4.2255	0.0116	*POLR2LP; CCHCR1*
13	rs16883089	6	73658053	C	T	3.8287	4.2847	0.0096	*KCNQ5*
14	rs10756704	9	15783816	A	G	−6.8602	−4.0591	0.0191	*CCDC171*
15	rs11222395	11	130794355	C	A	5.1305	3.8401	0.0349	*SNX19; RN7SL167P*
16	rs729438	14	104092789	A	G	4.2065	4.6938	0.0022	*KLC1; RP11-73M18.2*
17	rs3785632	17	15945608	C	T	−4.4051	−4.5014	0.0046	*TTC19; NCOR1*
18	rs8070454	17	38160754	T	C	−4.0026	−4.4159	0.0062	*PSMD3; RP11-387H17*
19	rs11654648	17	45913653	T	C	−3.5239	−4.3428	0.0143	*LRRC46*
20	rs7254272	19	4069119	A	G	−5.6994	−4.5576	0.0038	*ZBTB7A; MAP2K2*
20	rs350894	19	4115562	A	G	3.2379	4.3501	0.0330	*MAP2K2*
21	rs17721822	20	6469596	A	G	4.0971	−4.8002	0.0022	*CASC20*

Concordant lead SNPs identified by the conjFDR analysis and subsequently explored in PheWAS analyses are highlighted in grey.

**Figure 2.**
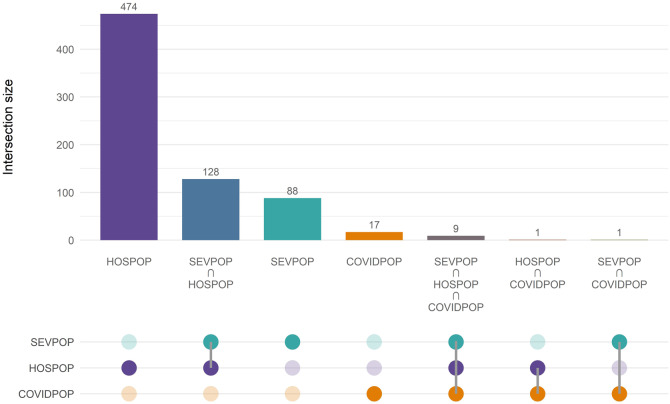
UpSet plot showing the overlap of SNPs jointly associated with BMI and COVID-19 phenotypes identified through conjFDR analysis. Each vertical bar represents the number of variants in a given intersection, while the connected dots below indicate the phenotype combinations involved. Bars representing intersections are coloured according to the combination of phenotypes involved. A total of nine variants were shared across all three phenotypes, whereas the largest overlap was observed between SEVPOP and HOSPOP. BMI; COVIDPOP, COVID versus population; HOSPOP, hospitalized COVID versus population; SEVPOP, very severe respiratory confirmed COVID versus population.

Considering all SNPs passing the conjFDR significance threshold (conjFDR < 0.05), nine variants (1.3% of the associations) were shared across all COVID-19 phenotypes and BMI: rs13107325-*SLC39A8*; rs1813006; rs2326788-*CASC20*; rs3213762; rs3859192*-GSDMA*; rs4794822; rs7502514-*MED24*; rs8070454-*PSMD3/ RP11-387H17*; rs8078723. Only one SNP was common between BMI and COVIDPOP and BMI and SEVPOP (rs3935280), and one between BMI and COVIDPOP and BMI and HOSPOP (rs4971079-*HMGN2P18/KRTCAP2*). The largest overlap occurred between BMI and SEVPOP and BMI and HOSPOP, which shared 128 variants (17.8% of the associations; figure 2). Permutation tests (100 000 permutations) confirmed that the observed overlaps were significantly greater than expected by chance (empirical *p* < 1 × 10^−5^).

Genes annotated to the lead SNPs were used as input for the functional enrichment analyses, being nine genes from the BMI versus COVIDPOP, 66 unique genes from the BMI versus HOSPOP and 34 genes from BMI versus SEVPOP (see tables 1–3 for the full list). Enrichment analyses revealed 43 significantly enriched Gene Ontology terms for the BMI and COVIDPOP comparison (table 4). No significant enrichment was observed for BMI and HOSPOP. In contrast, genes identified from the BMI and SEVPOP conjFDR analysis were significantly enriched in six gene ontology terms (table 4). No Reactome or KEGG pathways remained significant after multiple testing corrections for any of the phenotypes tested. All pathways with *p*-values lower than 0.05 before correction are reported in supplementary material, tables S4–S6.

**Table 4.** Functional enrichment: significant results.

*BMI and COVIDPOP*				
Term	*p*-value	*q*-value	number of overlapped genes	genes
**GO biological process**				
Protein N-linked Glycosylation (GO:0006487)	0.00021	**0.01427**	2/64	*KRTCAP2;SLC39A8*
Regulation of RNA Biosynthetic Process (GO:2001141)	0.00041	**0.01427**	3/465	*ZNF536;SLC39A8;LIMD1*
Protein Glycosylation (GO:0006486)	0.00107	**0.02498**	2/145	*KRTCAP2;SLC39A8*
Osteoblast Development (GO:0002076)	0.00210	**0.02808**	1/6	*LIMD1*
Manganese Ion Transport (GO:0006828)	0.00280	**0.02808**	1/8	*SLC39A8*
Zinc Ion Import Across Plasma Membrane (GO:0071578)	0.00280	**0.02808**	1/8	*SLC39A8*
Regulation of DNA-templated Transcription (GO:0006355)	0.00350	**0.02808**	4/2139	*MED24;ZNF536;SLC39A8;LIMD1*
Arginine Metabolic Process (GO:0006525)	0.00384	**0.02808**	1/11	*SLC39A8*
Iron Ion Transmembrane Transport (GO:0034755)	0.00384	**0.02808**	1/11	*SLC39A8*
Negative Regulation of Intracellular Signal Transduction (GO:1902532)	0.00439	**0.02808**	2/297	*SLC39A8;LIMD1*
P-body Assembly (GO:0033962)	0.00454	**0.02808**	1/13	*LIMD1*
Regulation of Gene Expression (GO:0010468)	0.00526	**0.02808**	3/1127	*ZNF536;SLC39A8;LIMD1*
Cellular Response to Cadmium Ion (GO:0071276)	0.00594	**0.02808**	1/17	*SLC39A8*
miRNA-mediated Gene Silencing by Inhibition of Translation (GO:0035278)	0.00594	**0.02808**	1/17	*LIMD1*
Intracellular Monoatomic Ion Homeostasis (GO:0006873)	0.00628	**0.02808**	1/18	*SLC39A8*
Leukocyte Adhesion to Vascular Endothelial Cell (GO:0061756)	0.00663	**0.02808**	1/19	*SLC39A8*
Bicarbonate Transport (GO:0015701)	0.00698	**0.02808**	1/20	*SLC39A8*
Regulatory ncRNA-mediated Post-Transcriptional Gene Silencing (GO:0035194)	0.00802	**0.02808**	1/23	*LIMD1*
Negative Regulation of Hippo Signaling (GO:0035331)	0.00802	**0.02808**	1/23	*LIMD1*
Zinc Ion Transmembrane Transport (GO:0071577)	0.00802	**0.02808**	1/23	*SLC39A8*
Zinc Ion Transport (GO:0006829)	0.00872	**0.02865**	1/25	*SLC39A8*
miRNA-mediated Post-Transcriptional Gene Silencing (GO:0035195)	0.00941	**0.02865**	1/27	*LIMD1*
Alpha-Amino Acid Metabolic Process (GO:1901605)	0.00941	**0.02865**	1/27	*SLC39A8*
Intracellular Zinc Ion Homeostasis (GO:0006882)	0.01011	**0.02948**	1/29	*SLC39A8*
Transition Metal Ion Transport (GO:0000041)	0.01080	**0.03024**	1/31	*SLC39A8*
Negative Regulation of Osteoblast Differentiation (GO:0045668)	0.01219	**0.03281**	1/35	*LIMD1*
Protein N-linked Glycosylation via Asparagine (GO:0018279)	0.01288	**0.03339**	1/37	*KRTCAP2*
Regulation of Hippo Signaling (GO:0035330)	0.01426	**0.03566**	1/41	*LIMD1*
Positive Regulation of Transcription Elongation by RNA Polymerase II (GO:0032968)	0.01599	**0.03860**	1/46	*MED24*
Positive Regulation of Transcription Initiation by RNA Polymerase II (GO:0060261)	0.01875	**0.04232**	1/54	*MED24*
Positive Regulation of DNA-templated Transcription, Elongation (GO:0032786)	0.01875	**0.04232**	1/54	*MED24*
Positive Regulation of DNA-templated Transcription Initiation (GO:2000144)	0.02047	**0.04232**	1/59	*MED24*
Regulation of Transcription Initiation by RNA Polymerase II (GO:0060260)	0.02047	**0.04232**	1/59	*MED24*
RNA Polymerase II Preinitiation Complex Assembly (GO:0051123)	0.02081	**0.04232**	1/60	*MED24*
Negative Regulation of Canonical NF-kappaB Signal Transduction (GO:0043124)	0.02116	**0.04232**	1/61	*SLC39A8*
Transcription Preinitiation Complex Assembly (GO:0070897)	0.02185	**0.04248**	1/63	*MED24*
**GO molecular function**				
Solute:Monoatomic Cation Symporter Activity (GO:0015294)	0.00350	**0.01813**	1/10	*SLC39A8*
Monoatomic Cation:Bicarbonate Symporter Activity (GO:0140410)	0.00419	**0.01813**	1/12	*SLC39A8*
Nuclear Vitamin D Receptor Binding (GO:0042809)	0.00454	**0.01813**	1/13	*MED24*
Bicarbonate Transmembrane Transporter Activity (GO:0015106)	0.00802	**0.01813**	1/23	*SLC39A8*
Zinc Ion Transmembrane Transporter Activity (GO:0005385)	0.00837	**0.01813**	1/24	*SLC39A8*
Nuclear Thyroid Hormone Receptor Binding (GO:0046966)	0.00907	**0.01813**	1/26	*MED24*
Transition Metal Ion Transmembrane Transporter Activity (GO:0046915)	0.01219	**0.02089**	1/35	*SLC39A8*
*BMI and SEVPOP*				
**GO biological process**				
Negative Regulation of Androgen Receptor Signaling Pathway (GO:0060766)	0.00008	**0.01554**	2/14	*NCOR1;ZBTB7A*
Regulation of Androgen Receptor Signaling Pathway (GO:0060765)	0.00038	**0.02619**	2/29	*NCOR1;ZBTB7A*
Negative Regulation of Intracellular Steroid Hormone Receptor Signaling Pathway (GO:0033144)	0.00043	**0.02619**	2/31	*NCOR1;ZBTB7A*
Regulation of Glycolytic Process (GO:0006110)	0.00080	**0.03614**	2/42	*NCOR1;ZBTB7A*
**GO Cellular Component**				
Microtubule (GO:0005874)	0.00091	**0.02993**	3/194	*MACF1;MAP2K2;BCL2L11*
Polymeric Cytoskeletal Fiber (GO:0099513)	0.00259	**0.04280**	3/280	*MACF1;MAP2K2;BCL2L11*

**Figure 3.**
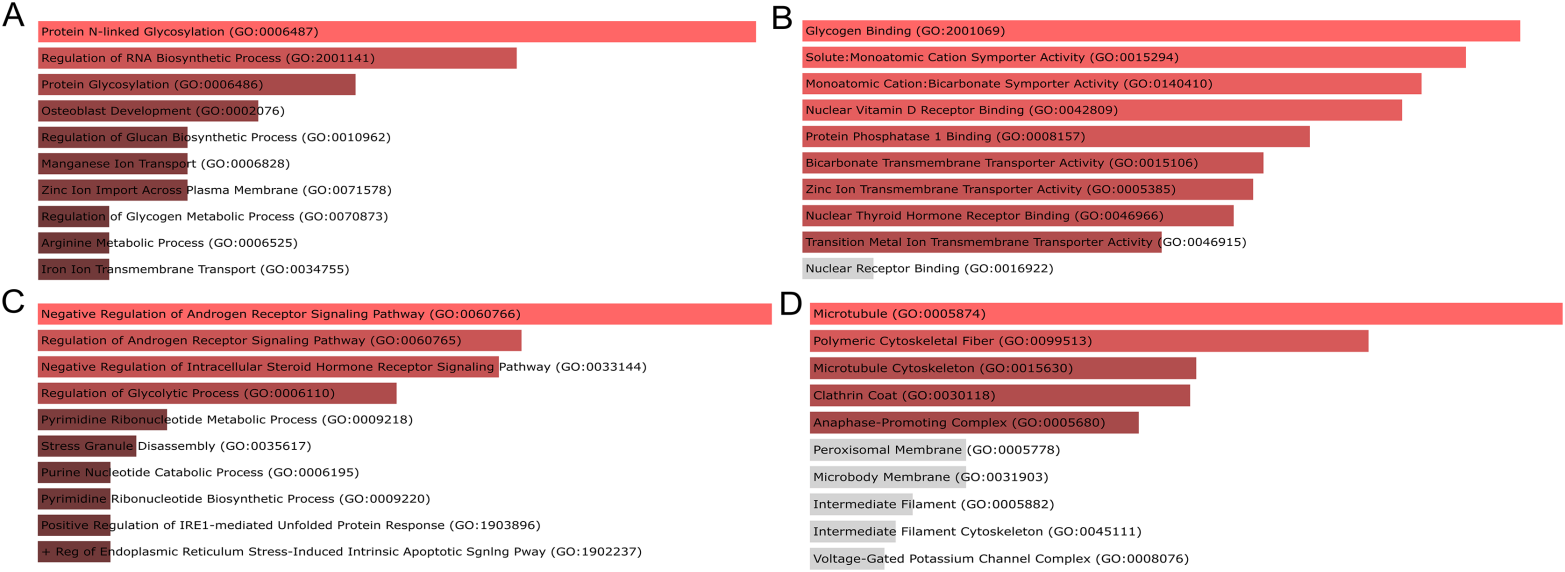
Bar graphs generated with EnrichR displaying the most significant gene ontology (GO) terms for each cross-trait analysis. Bar length represents the level of statistical significance. (A, B) Enrichment results for the BMI and COVIDPOP genes, showing biological process (A) and molecular function (B) categories. (C, D) Enrichment results for the BMI and SEVPOP, showing biological process (C) and cellular component (D) categories. Only significantly enriched terms (FDR-adjusted *q*-value < 0.05) are shown.

Bar graphs generated with EnrichR are presented in figure 3 to facilitate visualization of the most significant results. For BMI and COVIDPOP, the significant terms were mainly associated with biological processes (figure 3A) and molecular functions (figure 3B), including N-linked glycosylation, metal ion transport, transcriptional regulation and miRNA-mediated gene silencing. For BMI and SEVPOP, the enriched pathways involved biological processes (figure 3C) and cellular components (figure 3D), such as androgen receptor signaling, glycolytic process and cytoskeletal organization.

**Figure 4.**
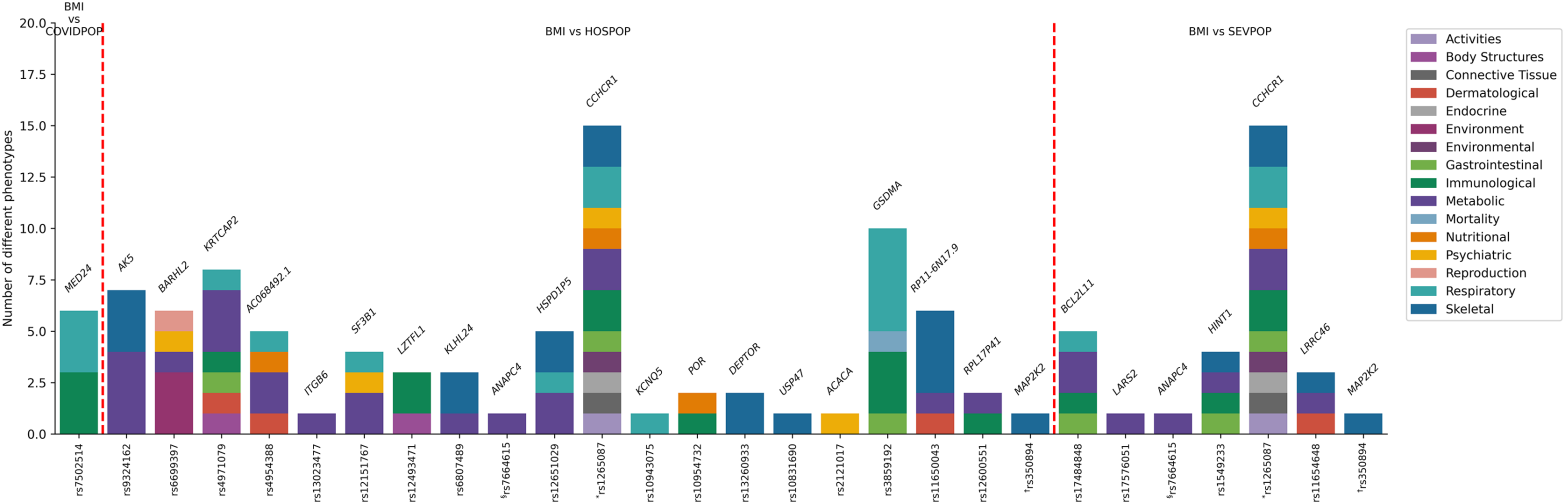
PheWAS results for the evaluated SNPs. Among the 35 lead and concordant SNPs analysed with pheWAS, only those contained in GWAS atlas are represented in the figure. Overall, 28 SNPs had data for previous associations in GWAS atlas. The plot displays significant associations across various phenotype domains. The height of each coloured segment represents the proportion of significant associations observed within that domain, allowing a comparative view of pleiotropic effects across different categories/domains. To avoid overrepresentation of domains in which several similar phenotypes were tested, we considered the number of distinct studies contributing to each domain, rather than the number of individual phenotypes. SNPs marked with the same symbol reflect associations in common between the groups. BMI; COVIDPOP, COVID versus population; HOSPOP, hospitalized COVID versus population; SEVPOP, very severe respiratory confirmed COVID versus population.

**Figure 5.**
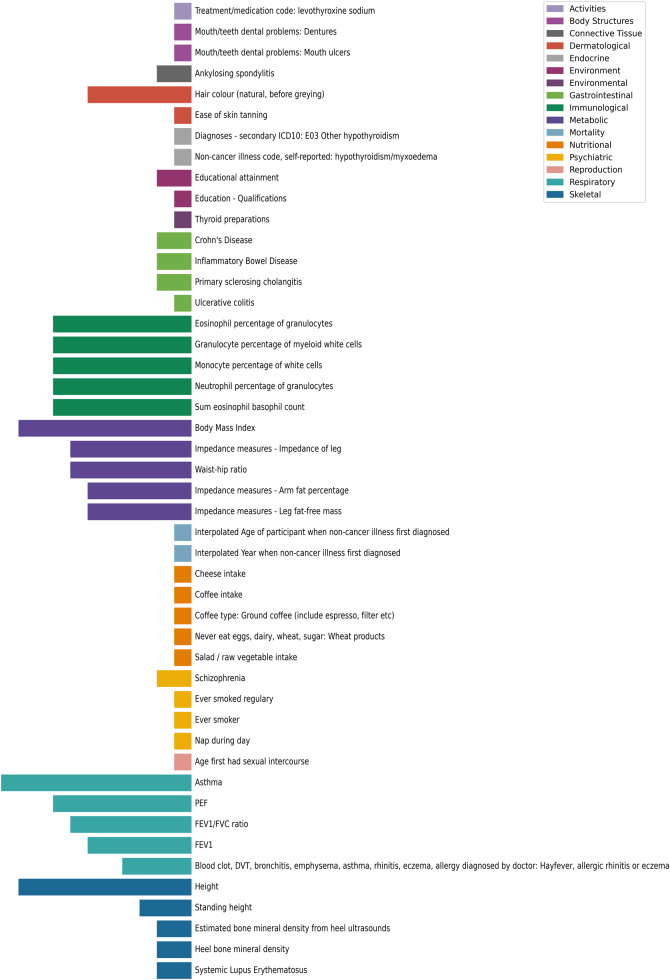
Top five most associated traits per PheWAS domain. Phenotypic traits were shown by domain (e.g. *Connective Tissue*, *metabolic*) to improve interpretability. For visualization purposes, highly similar or lateralized traits (e.g. ‘leg fat mass (left)’ and ‘leg fat mass (right)’) were merged into a single representative label (‘leg fat mass’). The size of each bar represents the total number of associations found by PheWAS.

Overall, the PheWAS results indicated pleiotropy among the SNPs evaluated, with previous GWAS identifying associations across a wide range of phenotypes grouped in several domains (figure 4). The domains most frequently observed were metabolic, skeletal, respiratory and immunological. From a phenotypic perspective, the traits with a higher number of different SNPs and/or associations were asthma (for respiratory domain), BMI and other traits related to adiposity (metabolic domain), height (skeletal domain) and traits related to white cell count (immunological domain). In figure 5, the five traits most frequently associated in each domain considered in PheWAS are represented.

## 4. Discussion

Conjunctional FDR (conjFDR) analysis revealed a substantial genetic overlap between BMI and the COVID-related phenotypes analysed. The number of associations varied across the three comparisons, suggesting that the shared biology with BMI differs among the COVID-19-related phenotypes. The analysis involving COVID-19 infection susceptibility yielded the fewest pleiotropic SNPs, despite this phenotype having the largest sample size. While the higher number of associations observed in the hospitalized phenotype compared to the very severe phenotype (which had the smallest sample size) may partly reflect differences in statistical power, this proportionality does not explain the markedly reduced overlap observed with the infection phenotype. Our findings suggest a biological explanation for an apparent clinical trend, since the literature provides limited evidence linking obesity to increased susceptibility to SARS-CoV-2 infection [24], indicating that the shared genetic architecture between obesity and COVID-19 is more tightly linked to mechanisms underlying disease severity than to initial susceptibility to infection.

In all three comparisons, the conjFDR approach identified independent pleiotropic variants, several of which were not previously reported as top hits in single-trait GWAS for either COVID-19 or obesity, suggesting novel pleiotropic regions. Specifically, two of the seven lead SNPs from the COVID-19 infection analysis, 26 of the 50 from the hospitalized COVID-19 analysis, and 7 of the 24 from the very severe phenotype represent novel associations uncovered by the cross-trait approach. These findings reinforce the power of conjFDR to identify pleiotropic and possibly causal variants to detect shared genetic components that may not reach genome-wide significance in single-phenotype GWAS.

Despite the substantial number of pleiotropic variants identified across all analyses, only nine of them (1.3%) were shared across the three COVID-19 phenotypes tested with BMI. Notably, the overlap between the infection phenotype (COVIDPOP) and either of the severity phenotypes (SEVPOP or HOSPOP) was minimal, with only one variant in common in each case. In contrast, the comparison between the two severity-related phenotypes yielded the largest intersection (128 SNPs and 17.8% jointly associated with BMI). This distribution supports the emerging evidence that the genetic architecture underlying susceptibility to SARS-CoV-2 infection is distinct from that associated with disease severity. Moreover, it reinforces that the genetic contribution shared between obesity and COVID-19 is specifically related to the severity of the disease.

Among the variants shared across all three COVID-19 phenotypes tested with BMI, a broadly pleiotropic profile is observed, with SNPs involved in metabolic and immune-related functions. The recurrence of such variants suggests broader biological processes potentially influencing both infection susceptibility and disease progression. The rs13107325 (*SLC39A8*) variant stands out for its wide-ranging effects, having been previously associated with Crohn's disease, schizophrenia, hypertension and reduced manganese levels [24–26]. This SNP appears to affect glycosylation and immune regulation by altering ZIP8-mediated manganese transport, thereby affecting multiple complex phenotypes [27]. Other shared variants, such as rs3859192 (*GSDMA*), have also been implicated in more than one metabolic and immune-related trait, including asthma, cardiovascular disease and white blood cell count [28,29]. Recent evidence shows that targeting N-linked glycans significantly inhibits viral entry, underscoring the critical role of host glycosylation in viral infectivity by sugar from a glycan shield on the SARS-CoV-2 spike [30]. The recurrence of these pleiotropic variants involved in metabolic and immunological functions reflects broader biological processes potentially influencing both infection and disease progression.

The two SNPs shared exclusively between the infection phenotype (COVIDPOP) and each of the severity-related phenotypes may reflect intermediate mechanisms linking susceptibility and disease progression. While rs3935280 (overlapping with SEVPOP) lacks functional characterization in the literature to date, rs4971079 (overlapping with HOSPOP) is associated with a range of chronic inflammatory conditions, including psoriasis and ulcerative colitis, suggesting a link to sustained or chronic low-grade inflammation [31]. The rs4971079 variant was also identified in the cross-trait GWAS by Chen *et al.*, which aimed to detect pleiotropic loci between BMI and hospitalized COVID-19 using a distinct methodological approach [17]. This convergence reinforces the pleiotropic relevance of the locus and exemplifies how different analytical strategies can uncover complementary aspects of the shared genetic architecture between obesity and COVID-19 outcomes.

Among the 128 variants shared exclusively between the two severity-related phenotypes (SEVPOP and HOSPOP), those previously reported in GWAS were consistently associated with systemic inflammation and immune activation traits, such as C-reactive protein levels (rs17391694, rs178810, rs178826), Cystatin C levels (rs17391694, rs11806197) and eosinophil percentage (rs11880023), or were linked to pulmonary traits, including chronic obstructive pulmonary disease-related biomarkers (rs1265093), lung function measures and peak expiratory flow (rs13206405) [32–38]. Unlike the more discrete or early-stage immune signatures observed in intermediate overlap, these loci probably converge on pathways of immune hyperactivation and tissue damage that characterize severe disease.

Interestingly, although the COVIDPOP phenotype showed the fewest associations in the conjFDR analysis, it yielded the largest number of enriched pathways (*n* = 43). This apparent discrepancy suggests that, despite fewer overlapping variants, those shared between BMI and COVID-19 susceptibility may exert broader and more constitutive biological effects, potentially influencing a diverse array of fundamental processes across multiple systems. The enriched processes, including N-linked protein glycosylation, metal ion transport (manganese, zinc, iron), transcriptional regulation and miRNA-mediated gene silencing, play roles in cellular stress response, immune modulation and metabolic control. The presence of pathways such as leukocyte adhesion to endothelial cells and negative regulation of NF-κB signaling further supports the idea of a generalized, yet biologically relevant, interaction between obesity and the initial immune response to infection.

In SARS-CoV-2 infection, endothelial activation with upregulated ICAM-1/VCAM-1 promotes leukocyte adhesion and immunothrombosis, hallmarks of COVID-19-induced endotheliitis documented in lung autopsies that link vascular barrier dysfunction to the host's initial antiviral response [39]. Simultaneously, obesity skews early innate responses via adipokine imbalance (reduced adiponectin/leptin resistance), weakening negative feedback over NF-κB while SARS-CoV-2 proteins actively drive NF-κB and blunt interferon; this combination fosters a pro-adhesive, pro-inflammatory microenvironment that facilitates viral replication and escalation to severe disease [40,41]. These findings may reflect a less specific and systemic genetic link between obesity and COVID-19 infection.

The absence of enriched terms in the hospitalization phenotype (HOSPOP) analysis, despite its greater number of conjFDR hits, may reflect the diffuse nature of this phenotype. Although related to disease severity, hospitalization encompasses a wide clinical spectrum, from brief admissions with full recovery to prolonged intensive care or even death. This phenotypic heterogeneity could dilute consistent biological signals, as the underlying genetic associations may be distributed across diverse and non-overlapping pathways. Such dispersion may prevent the convergence of hits within specific annotated pathways, despite their individual contribution to disease severity. Moreover, the characteristics of the gene set itself, particularly a relatively large number of associated genes, may also contribute to signal dilution, further hindering the detection of significant enrichment. Alternatively, it is also possible that these variants are involved in mechanisms not currently represented in pathway annotation databases, and therefore, their functional convergence remains undetected by available enrichment tools.

In contrast, the six enriched pathways identified in the very severe COVID-19 phenotype, which include regulation of androgen receptor signaling, glycolytic processes and cytoskeletal components (e.g. microtubules), may reflect a more specific set of biological mechanisms consistent with severe disease manifestations, involving metabolic stress, hormone signaling and cell structure integrity. Notably, the involvement of androgen signaling aligns with clinical observations of greater COVID-19 severity in men, a trend also reported in other studies, including independent clinical data from our group [42]. In the context of virus biology, androgen receptor-driven upregulation of ACE2 and TMPRSS2 enhances SARS-CoV-2 cellular entry and replication [43], while a parallel shift toward HIF-1α-mediated glycolysis in infected monocytes fuels viral proliferation and exacerbates inflammatory responses [44]. This convergence suggests that sex-specific hormonal pathways may contribute to differential genetic vulnerability in severe outcomes.

The PheWAS analysis showed that the evaluated SNPs displayed a consistent pleiotropic profile, with all of them being associated with more than one phenotype and their prior associations spanning a wide range of biological domains. Notably, the most recurrent domains (metabolic, immunological, respiratory and skeletal) reflect biological processes previously implicated in our pathway analyses, such as metabolic stress, immune dysregulation, and impaired lung and tissue repair capacity. As expected, traits related to adiposity, such as waist-hip ratio, arm fat percentage and leg fat-free mass, were among the most recurrent.

Other traits, including asthma and lung function, height and white blood cell counts, also emerged frequently among the top SNPs. Broadly examining the identified traits, many converge on inflammatory processes: asthma, white blood cell traits, systemic lupus erythematosus, Crohn's disease, inflammatory bowel disease and ankylosing spondylitis. Still, our findings also highlight phenotypes beyond this axis, such as height, heel bone mineral density and schizophrenia, which warrant further investigation. These patterns reinforce the notion that the genetic link between obesity and COVID-19 severity is rooted in broad and interconnected biological mechanisms rather than isolated pathways. Among these, inflammatory imbalance emerges as a central axis, but the diversity of associated traits suggests a complex architecture that may involve multiple physiological systems.

A limitation of this study is the restriction of our analyses to summary-level data from individuals of European ancestry, which bounds the generalizability of our findings to other populations. The exclusion of non-European datasets was necessary to avoid population stratification bias, but it reduces the ability to capture ancestry-specific pleiotropic signals, especially considering the known disparities in COVID-19 outcomes across populations. As GWAS summary statistics are aggregated from multiple cohorts with heterogeneous study designs and phenotype definitions, another limitation is that phenotype characterization may be less precise than in single-cohort analyses. In addition, the COVID-19 GWAS summary statistics do not include individual-level phenotypic information such as BMI, sex or other clinical covariates, precluding stratified analyses to evaluate whether some of the shared loci identified could be influenced by differences in obesity status among COVID-19 cases or by sex-specific genetic effects across the traits analysed.

Another consideration is the imbalance in the summary statistics of the traits analysed, as the GWAS dataset for BMI had a considerably larger sample size compared to the COVID-19 datasets, which may have affected the detection of shared signals. In addition, while the PheWAS approach provided a broader phenotypic context, it relied on previously published associations and is subject to the limitations of the original studies. Finally, our study focused on BMI as a continuous trait to represent obesity; although BMI is widely used as a proxy for obesity in genetic studies, it does not capture differences in body fat distribution or metabolic heterogeneity. Other adiposity-related traits, such as waist-to-hip ratio, as well as the concept of metabolically healthy obesity, may provide complementary insights into the relationship between adiposity and COVID-19 outcomes. Future work may investigate whether different measures of adiposity yield distinct pleiotropic patterns when jointly analysed with COVID-19 outcomes.

Our study reveals a substantial and biologically meaningful genetic overlap between BMI and COVID-19 severity, mediated through pleiotropic loci associated with immune, metabolic and respiratory functions. The identification of shared variants and enriched pathways supports the hypothesis that obesity and severe COVID-19 converge on common biological mechanisms, particularly those involving inflammatory regulation and systemic stress responses. By integrating genome-wide data through a hypothesis-free pleiotropy-driven approach, this study advances our understanding of how host genetics may shape the interplay between pre-existing metabolic risk and severe infectious diseases. These insights offer a framework for future research aimed at disentangling shared etiological pathways and may inform preventive strategies or therapeutic targets that transcend the boundaries of individual diseases.

## Data accessibility

This study used publicly available genome-wide association summary statistics from previously published datasets. COVID-19 GWAS summary data were obtained from the COVID-19 Host Genetics Initiative (release 7; https://www.covid19hg.org/results/r7/), and BMI GWAS summary data were obtained from the GIANT consortium (https://portals.broadinstitute.org/collaboration/giant/index.php/GIANT_consortium_data_files). All analyses were performed using these open-access datasets, and no new data were generated. The scripts used for data processing and statistical analysis are available from the corresponding author.
